# 5-Amino-3-carb­oxy-1*H*-1,2,4-triazol-4-ium nitrate monohydrate

**DOI:** 10.1107/S1600536812011154

**Published:** 2012-03-21

**Authors:** Fadila Berrah, Rafika Bouchene, Sofiane Bouacida, Thierry Roisnel

**Affiliations:** a Laboratoire de Chimie Appliquée et Technologie des Matériaux LCATM, Université Larbi Ben M’Hidi, 04000 Oum El Bouaghi, Algeria; b Unité de Recherche de Chimie de l’Environnement et Moléculaire Structurale, CHEMS, Faculté des Sciences Exactes, Université Mentouri Constantine 25000, Algeria; c Centre de difractométrie X, UMR 6226 CNRS Unité Sciences Chimiques de Rennes, Université de Rennes I, 263 Avenue du Général Leclerc, 35042 Rennes, France

## Abstract

The two-dimensional crystal packing of the title compound, C_3_H_5_N_4_O_2_^+^·NO_2_^−^·H_2_O, results from the stacking of well separated layers (*i.e.* with nothing between the layers) parallel to the (-113) plane in which adjacent cations adopt a head-to-head arrangement such that two –COOH groups are linked *via* two water mol­ecules (the water O atom behaves simultaneously as donor and acceptor of hydrogen bonds) and two –NH_2_ groups are linked through two nitrate anions. This arrangement leads to alternating hydro­philic and hydro­phobic zones in which O—H⋯O and N—H⋯O hydrogen bonds, respectively, are observed.

## Related literature

For properties of 1,2,4-triazoles, see: Ouakkaf *et al.* (2011 ▶). For related structures, see: Fernandes *et al.* (2011 ▶); Berrah *et al.* (2011*a* ▶,*b* ▶); Jebas *et al.* (2006 ▶).
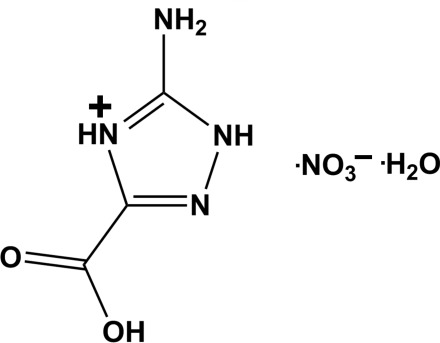


## Experimental

### 

#### Crystal data

C_3_H_5_N_4_O_2_^+^·NO_3_^−^·H_2_O
*M* *_r_* = 209.14
Triclinic, 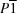

*a* = 4.9934 (13) Å
*b* = 6.7454 (17) Å
*c* = 12.446 (3) Å
α = 97.572 (12)°
β = 100.524 (13)°
γ = 98.933 (13)°
*V* = 401.60 (18) Å^3^
*Z* = 2
Mo *K*α radiation
μ = 0.17 mm^−1^
*T* = 150 K
0.42 × 0.2 × 0.11 mm

#### Data collection

Bruker APEXII diffractometer
Absorption correction: multi-scan (*SADABS*; Sheldrick, 2002 ▶) *T*_min_ = 0.863, *T*_max_ = 0.982
4012 measured reflections
1821 independent reflections
1563 reflections with *I* > 2σ(*I*)
*R*_int_ = 0.040

#### Refinement

*R*[*F* ^2^ > 2σ(*F* ^2^)] = 0.040
*wR*(*F* ^2^) = 0.109
*S* = 1.03
1821 reflections
134 parameters
H atoms treated by a mixture of independent and constrained refinement
Δρ_max_ = 0.35 e Å^−3^
Δρ_min_ = −0.29 e Å^−3^

### 

Data collection: *APEX2* (Bruker, 2006 ▶); cell refinement: *SAINT* (Bruker, 2006 ▶); data reduction: *SAINT*; program(s) used to solve structure: *SIR2002* (Burla *et al.*, 2005 ▶); program(s) used to refine structure: *SHELXL97* (Sheldrick, 2008 ▶); molecular graphics: *ORTEP-3 for Windows* (Farrugia, 1997 ▶) and *DIAMOND* (Brandenburg & Berndt, 2001 ▶); software used to prepare material for publication: *WinGX* (Farrugia, 1999 ▶).

## Supplementary Material

Crystal structure: contains datablock(s) global, I. DOI: 10.1107/S1600536812011154/pv2522sup1.cif

Structure factors: contains datablock(s) I. DOI: 10.1107/S1600536812011154/pv2522Isup2.hkl

Supplementary material file. DOI: 10.1107/S1600536812011154/pv2522Isup3.cml

Additional supplementary materials:  crystallographic information; 3D view; checkCIF report

## Figures and Tables

**Table 1:** Hydrogen-bond geometry (Å, °)

*D*—H⋯*A*	*D*—H	H⋯*A*	*D*⋯*A*	*D*—H⋯*A*
O5—H5⋯O1*W*	0.82	1.72	2.5210 (17)	166
O1*W*—H2*W*⋯O4^i^	0.84 (3)	1.97 (3)	2.7985 (18)	166 (2)
O1*W*—H1*W*⋯N3^ii^	0.86 (2)	2.05 (3)	2.9011 (19)	172 (2)
N5—H5*B*⋯O2^iii^	0.86	2.04	2.8352 (18)	154
N2—H2⋯O1^iii^	0.86	2.02	2.8790 (17)	178
N4—H4⋯O1	0.86	2.06	2.9112 (18)	171

Symmetry codes: (i) 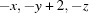; (ii) 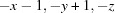; (iii) 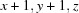.

**Fig. 1.**
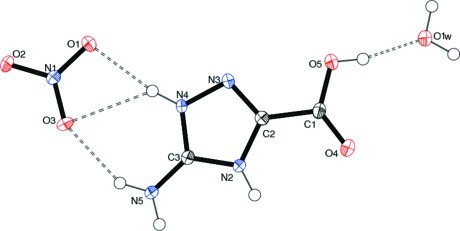
An asymmetric unit of the title compound with the atomic labelling scheme. Displacement are drawn at the 50% probability level. Hydrogen bonds are shown as dashed lines.

**Fig. 2.**
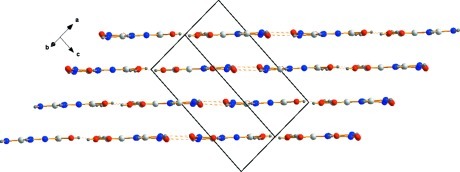
A two-dimensional network of the title compound viewed along the [1–10] direction. Hydrogen bonds are shown as dashed lines.

**Fig. 3.**
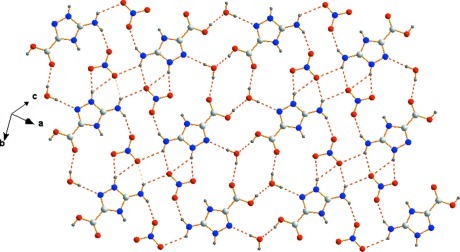
A view of the title compound parallel to the (-113 ) plane of the planar infinite layer showing alternating hydrophilic and hydrophobic zones involving O—H···O and N—H···O hydrogen bonds, respectively; hydrogen bonds are shown as dashed lines.

## supplementary crystallographic information

### Comment

Following our on-going interest on crystal structures of hybrid compounds established by hydrogen bonds and in attempts to clarify anion substitution influence upon hydrogen bonding patterns, we have undertaken synthesis of new compounds using 1,2,4-triazol derivatives and various inorganic acids (Ouakkaf *et al.*, 2011). In this article, we report the preparations and crystal structure of the title compound.

The asymmetric unit of the title compound contains a cation, an anion and a water molecule linked by O—H···O and N—H···O hydrogen bonds (Fig.1.) The geometry of the triazole planar ring is similar to that seen in related compounds (Fernandes *et al.*, 2011; Ouakkaf *et al.*, 2011); it exhibits a short distance of 1.3023 (19) Å showing the double-bond formed between atoms C2 and N3, two intermediat bonds (1.3443 (18) and 1.3529 (19) Å) associated with a delocalized double bond (N4 ≐C3 ≐N2), and two long distances 1.3698 (19) and 1.3779 (18) Å related to the single bonds C2—N2 and N3—N4, respectively.

The two-dimensional network of the title compound results from the stacking of well separated planar layers parallel to (-113) plane (Fig. 2); analogous networks have been observed in other nitrate compounds (Berrah *et al.*, 2011*a*,*b*; Jebas *et al.*, 2006). In each layer, the adjacent cations are oriented in a head to head configuration in such a manner that two –COOH groups are linked *via* two water molecules (H_2_O behaves simultaneously as donor and acceptor of hydrogen bonds) and two –NH_2_groups are linked through two nitrate anions (Fig. 3 and Table 1). This arrangement leads to an alternating hydrophilic and hydrophobic zones where O—H···O and N—H···O H-bonds are observed, respectively.

### Experimental

Colourless crystals of the title compound were grown by slow evaporation of water-methanol (1:1) solution of 5-amino-1,2,4-triazol-1*H*-3-carboxylic acid hydrate and nitric acid in a 1:1 stoichiometric ratio.

### Refinement

The H atoms of the water molecule were located from a difference Fourier map and were refined with *U*_iso_(H) = 1.5*U*_eq_(O). The remaining H atoms were located from differnce Fourier maps but introduced in calculated positions and treated as riding on their parent atoms with O—H = 0.82 Å and N—H = 0.86 Å with *U*_iso_(H) = 1.2*U*_eq_(N) and 1.5*U*_eq_(O).

### Crystal data

**Table:**

C_3_H_5_N_4_O_2_^+^·NO_3_^−^·H_2_O	*Z* = 2
*M**_r_* = 209.14	*F*(000) = 216
Triclinic, *P*1	*D*_x_ = 1.729 Mg m^−^^3^
*a* = 4.9934 (13) Å	Mo *K*α radiation, λ = 0.71073 Å
*b* = 6.7454 (17) Å	Cell parameters from 1584 reflections
*c* = 12.446 (3) Å	θ = 3.4–27.4°
α = 97.572 (12)°	µ = 0.17 mm^−^^1^
β = 100.524 (13)°	*T* = 150 K
γ = 98.933 (13)°	Stick, colourless
*V* = 401.60 (18) Å^3^	0.42 × 0.2 × 0.11 mm

### Data collection

**Table:**

Bruker APEXII diffractometer	1563 reflections with *I* > 2σ(*I*)
Graphite monochromator	*R*_int_ = 0.040
CCD rotation images, thin slices scans	θ_max_ = 27.5°, θ_min_ = 3.1°
Absorption correction: multi-scan (*SADABS*; Sheldrick, 2002)	*h* = −6→6
*T*_min_ = 0.863, *T*_max_ = 0.982	*k* = −6→8
4012 measured reflections	*l* = −16→15
1821 independent reflections	

### Refinement

**Table:**

Refinement on *F*^2^	Primary atom site location: structure-invariant direct methods
Least-squares matrix: full	Secondary atom site location: difference Fourier map
*R*[*F*^2^ > 2σ(*F*^2^)] = 0.040	Hydrogen site location: inferred from neighbouring sites
*wR*(*F*^2^) = 0.109	H atoms treated by a mixture of independent and constrained refinement
*S* = 1.03	*w* = 1/[σ^2^(*F*_o_^2^) + (0.0535*P*)^2^ + 0.0973*P*] where *P* = (*F*_o_^2^ + 2*F*_c_^2^)/3
1821 reflections	(Δ/σ)_max_ < 0.001
134 parameters	Δρ_max_ = 0.35 e Å^−^^3^
0 restraints	Δρ_min_ = −0.29 e Å^−^^3^

### Special details

**Table:**

Geometry. All e.s.d.'s (except the e.s.d. in the dihedral angle between two l.s. planes) are estimated using the full covariance matrix. The cell e.s.d.'s are taken into account individually in the estimation of e.s.d.'s in distances, angles and torsion angles; correlations between e.s.d.'s in cell parameters are only used when they are defined by crystal symmetry. An approximate (isotropic) treatment of cell e.s.d.'s is used for estimating e.s.d.'s involving l.s. planes.
Refinement. Refinement of *F*^2^ against ALL reflections. The weighted *R*-factor *wR* and goodness of fit *S* are based on *F*^2^, conventional *R*-factors *R* are based on *F*, with *F* set to zero for negative *F*^2^. The threshold expression of *F*^2^ > σ(*F*^2^) is used only for calculating *R*-factors(gt) *etc*. and is not relevant to the choice of reflections for refinement. *R*-factors based on *F*^2^ are statistically about twice as large as those based on *F*, and *R*- factors based on ALL data will be even larger.

### Fractional atomic coordinates and isotropic or equivalent isotropic displacement parameters (Å^2^)

**Table:**

	*x*	*y*	*z*	*U*_iso_*/*U*_eq_	
O1	−0.1877 (2)	−0.15602 (16)	0.31865 (9)	0.0218 (3)	
N3	−0.0716 (3)	0.32704 (19)	0.20042 (11)	0.0174 (3)	
O4	0.2001 (2)	0.80430 (17)	0.13489 (10)	0.0241 (3)	
O5	−0.2192 (2)	0.59982 (18)	0.06811 (10)	0.0234 (3)	
H5	−0.2505	0.6888	0.0311	0.035*	
O1W	−0.3752 (3)	0.82557 (19)	−0.06786 (10)	0.0272 (3)	
H2W	−0.319 (5)	0.946 (4)	−0.0762 (18)	0.041*	
H1W	−0.544 (5)	0.791 (3)	−0.1043 (19)	0.041*	
N5	0.5272 (3)	0.2985 (2)	0.39298 (11)	0.0219 (3)	
H5A	0.4999	0.185	0.4172	0.026*	
H5B	0.6829	0.3811	0.4162	0.026*	
O2	−0.0623 (2)	−0.34292 (17)	0.44188 (10)	0.0284 (3)	
O3	0.2143 (2)	−0.06970 (17)	0.43144 (10)	0.0262 (3)	
N2	0.3435 (2)	0.51522 (18)	0.27273 (10)	0.0158 (3)	
H2	0.4806	0.6154	0.2854	0.019*	
N4	0.0781 (3)	0.23345 (19)	0.27646 (10)	0.0167 (3)	
H4	0.0176	0.1178	0.2937	0.02*	
N1	−0.0104 (3)	−0.18948 (19)	0.39831 (10)	0.0171 (3)	
C3	0.3308 (3)	0.3458 (2)	0.32034 (12)	0.0155 (3)	
C2	0.0952 (3)	0.4958 (2)	0.20038 (12)	0.0165 (3)	
C1	0.0296 (3)	0.6523 (2)	0.12995 (13)	0.0175 (3)	

### Atomic displacement parameters (Å^2^)

**Table:**

	*U*^11^	*U*^22^	*U*^33^	*U*^12^	*U*^13^	*U*^23^
O1	0.0199 (5)	0.0212 (6)	0.0216 (6)	0.0009 (4)	−0.0034 (4)	0.0088 (5)
N3	0.0166 (6)	0.0160 (6)	0.0191 (7)	0.0023 (5)	−0.0004 (5)	0.0077 (5)
O4	0.0237 (6)	0.0185 (6)	0.0289 (6)	−0.0007 (5)	0.0009 (5)	0.0109 (5)
O5	0.0219 (6)	0.0198 (6)	0.0261 (6)	0.0013 (5)	−0.0043 (5)	0.0114 (5)
O1W	0.0219 (6)	0.0221 (6)	0.0343 (7)	−0.0020 (5)	−0.0057 (5)	0.0159 (5)
N5	0.0150 (6)	0.0186 (7)	0.0293 (8)	−0.0034 (5)	−0.0034 (5)	0.0123 (6)
O2	0.0274 (6)	0.0189 (6)	0.0339 (7)	−0.0075 (5)	−0.0047 (5)	0.0156 (5)
O3	0.0183 (6)	0.0212 (6)	0.0334 (7)	−0.0073 (5)	−0.0039 (5)	0.0105 (5)
N2	0.0135 (6)	0.0134 (6)	0.0190 (6)	−0.0007 (5)	0.0002 (5)	0.0060 (5)
N4	0.0149 (6)	0.0150 (6)	0.0195 (6)	0.0004 (5)	−0.0011 (5)	0.0095 (5)
N1	0.0166 (6)	0.0139 (6)	0.0195 (7)	−0.0002 (5)	0.0015 (5)	0.0047 (5)
C3	0.0153 (7)	0.0134 (7)	0.0177 (7)	0.0016 (5)	0.0023 (6)	0.0045 (6)
C2	0.0151 (7)	0.0153 (7)	0.0182 (7)	0.0021 (5)	0.0008 (6)	0.0042 (6)
C1	0.0201 (7)	0.0136 (7)	0.0187 (7)	0.0022 (6)	0.0031 (6)	0.0053 (6)

### Geometric parameters (Å, º)

**Table:**

O1—N1	1.2720 (16)	N5—H5B	0.86
N3—C2	1.3023 (19)	O2—N1	1.2443 (16)
N3—N4	1.3779 (18)	O3—N1	1.2426 (16)
O4—C1	1.2139 (18)	N2—C3	1.3529 (19)
O5—C1	1.3051 (19)	N2—C2	1.3698 (19)
O5—H5	0.82	N2—H2	0.86
O1W—H2W	0.84 (3)	N4—C3	1.3443 (18)
O1W—H1W	0.86 (2)	N4—H4	0.86
N5—C3	1.3155 (19)	C2—C1	1.495 (2)
N5—H5A	0.86		
			
C2—N3—N4	103.98 (12)	O3—N1—O2	120.44 (13)
C1—O5—H5	109.5	O3—N1—O1	119.79 (12)
H2W—O1W—H1W	107 (2)	O2—N1—O1	119.77 (12)
C3—N5—H5A	120	N5—C3—N4	126.95 (13)
C3—N5—H5B	120	N5—C3—N2	127.13 (13)
H5A—N5—H5B	120	N4—C3—N2	105.91 (12)
C3—N2—C2	106.57 (12)	N3—C2—N2	112.16 (13)
C3—N2—H2	126.7	N3—C2—C1	124.96 (14)
C2—N2—H2	126.7	N2—C2—C1	122.89 (13)
C3—N4—N3	111.38 (12)	O4—C1—O5	128.33 (15)
C3—N4—H4	124.3	O4—C1—C2	120.16 (14)
N3—N4—H4	124.3	O5—C1—C2	111.50 (13)
			
C2—N3—N4—C3	−0.23 (16)	C3—N2—C2—N3	0.52 (17)
N3—N4—C3—N5	−178.18 (15)	C3—N2—C2—C1	−179.10 (13)
N3—N4—C3—N2	0.55 (16)	N3—C2—C1—O4	−179.23 (15)
C2—N2—C3—N5	178.10 (15)	N2—C2—C1—O4	0.3 (2)
C2—N2—C3—N4	−0.62 (15)	N3—C2—C1—O5	0.5 (2)
N4—N3—C2—N2	−0.18 (16)	N2—C2—C1—O5	−179.93 (13)
N4—N3—C2—C1	179.43 (14)		

### Hydrogen-bond geometry (Å, º)

**Table:**

*D*—H···*A*	*D*—H	H···*A*	*D*···*A*	*D*—H···*A*
O5—H5···O1*W*	0.82	1.72	2.5210 (17)	166
O1*W*—H2*W*···O4^i^	0.84 (3)	1.97 (3)	2.7985 (18)	166 (2)
O1*W*—H1*W*···N3^ii^	0.86 (2)	2.05 (3)	2.9011 (19)	172 (2)
N5—H5*A*···O3	0.86	2.1	2.8672 (18)	148
N5—H5*A*···O3^iii^	0.86	2.44	3.0498 (19)	129
N5—H5*B*···O2^iv^	0.86	2.04	2.8352 (18)	154
N5—H5*B*···O2^iii^	0.86	2.41	3.0060 (18)	127
N2—H2···O1^iv^	0.86	2.02	2.8790 (17)	178
N4—H4···O1	0.86	2.06	2.9112 (18)	171
N4—H4···O3	0.86	2.42	3.0590 (18)	132
N4—H4···N1	0.86	2.59	3.4099 (19)	160

Symmetry codes: (i) −*x*, −*y*+2, −*z*; (ii) −*x*−1, −*y*+1, −*z*; (iii) −*x*+1, −*y*, −*z*+1; (iv) *x*+1, *y*+1, *z*.
